# Capturing heart valve development with Gene Ontology

**DOI:** 10.3389/fgene.2023.1251902

**Published:** 2023-10-17

**Authors:** Saadullah H. Ahmed, Alexander T. Deng, Rachael P. Huntley, Nancy H. Campbell, Ruth C. Lovering

**Affiliations:** ^1^ Functional Gene Annotation, Pre-clinical and Fundamental Science, Institute of Cardiovascular Science, University College London, London, United Kingdom; ^2^ Department of Clinical Genetics, Guy’s and St Thomas’s NHS Foundation Trust, London, United Kingdom; ^3^ SciBite Limited, BioData Innovation Centre, Wellcome Genome Campus, Hinxton, Cambridge, United Kingdom; ^4^ Healx, Cambridge, United Kingdom

**Keywords:** Gene Ontology, heart valve development, functional analysis, transcriptomics, proteomics, genomics, bioinformatics

## Abstract

**Introduction:** The normal development of all heart valves requires highly coordinated signaling pathways and downstream mediators. While genomic variants can be responsible for congenital valve disease, environmental factors can also play a role. Later in life valve calcification is a leading cause of aortic valve stenosis, a progressive disease that may lead to heart failure. Current research into the causes of both congenital valve diseases and valve calcification is using a variety of high-throughput methodologies, including transcriptomics, proteomics and genomics. High quality genetic data from biological knowledge bases are essential to facilitate analyses and interpretation of these high-throughput datasets. The Gene Ontology (GO, http://geneontology.org/) is a major bioinformatics resource used to interpret these datasets, as it provides structured, computable knowledge describing the role of gene products across all organisms. The UCL Functional Gene Annotation team focuses on GO annotation of human gene products. Having identified that the GO annotations included in transcriptomic, proteomic and genomic data did not provide sufficient descriptive information about heart valve development, we initiated a focused project to address this issue.

**Methods:** This project prioritized 138 proteins for GO annotation, which led to the curation of 100 peer-reviewed articles and the creation of 400 heart valve development-relevant GO annotations.

**Results:** While the focus of this project was heart valve development, around 600 of the 1000 annotations created described the broader cellular role of these proteins, including those describing aortic valve morphogenesis, BMP signaling and endocardial cushion development. Our functional enrichment analysis of the 28 proteins known to have a role in bicuspid aortic valve disease confirmed that this annotation project has led to an improved interpretation of a heart valve genetic dataset.

**Discussion:** To address the needs of the heart valve research community this project has provided GO annotations to describe the specific roles of key proteins involved in heart valve development. The breadth of GO annotations created by this project will benefit many of those seeking to interpret a wide range of cardiovascular genomic, transcriptomic, proteomic and metabolomic datasets.

## 1 Introduction

Heart valve development involves complex interactions between a multitude of signaling pathways initiated by locational cues. Changes to these cues or the functionality of the components within or downstream of these signaling pathways often leads to valve malformation and disease. Any imperfections in the very early stages of embryonic heart development have the potential to cause defects in the subsequent heart formation. However, for the heart valves, it is the development of the outflow tract and endocardial cushions, as well as the process of epithelial–mesenchymal transition (EMT) and the signaling pathways directly involved in the process of valve formation that appear to be key to the development of these structures (Bonachea et al., 2014; Williams et al., 2019; Bravo-Jaimes and Prakash, 2020). In addition, there is growing evidence that abnormal embryonic heart valve development can increase the risk of valve disease later in life, with the signaling pathways that control early valve development playing a role in valve calcification (Wirrig and Yutzey, 2014). With more than 2% of the population suffering from heart valve disease, understanding the molecular mechanisms has the potential to inform future therapies (Albanese et al., 2017; Williams et al., 2019).

While approximately 400 genes are thought to be associated with congenital heart disease only around 40 congenital heart valve disease-risk genes have been identified (Wu et al., 2017; Williams et al., 2019). Many of these genes have been identified through human and mouse genetic studies as well as next-generation sequencing (NGS). As aortic valve defects, such as bicuspid aortic valve (BAV), are the most common congenital heart malformations (Bonachea et al., 2014) it is important to ensure that the genes required for appropriate formation of this valve are fully described in genetic databases. These genes encode proteins important in two key processes: 1) signaling pathways and regulation of transcription and 2) structural composition and organization of valve extracellular matrix (Hinton and Yutzey, 2011).

One of the main cellular processes that takes place during valvulogenesis is endothelial to mesenchymal transformation (Combs and Yutzey, 2009). This process gives rise to a population of highly proliferative and migratory valve precursor cells. Although, many signaling pathways have been found to be associated with EMT, a relatively small set of highly evolutionarily conserved signaling pathways involving the notch receptor (NOTCH), transforming growth factor-beta (TGFB)/bone morphogenic protein (BMP), and WNT family members, as well as the pathways initiated by vascular endothelial growth factor A (VEGFA), stand out as having critical roles during valve development (Combs and Yutzey, 2009; Williams et al., 2019). For instance, NOTCH and BMP2 regulate activities of the transcription factors, SNAI1 and 2, which are required for EMT to take place during cushion formation in the atrioventricular canal (AVC) (Luna-Zurita et al., 2010). All of these signaling pathways are inter-connected with one another to form a complex genetic network regulating the development of a variety of different cell types. For example, the majority of growth factor receptors responsible for the initiation of EMT are NOTCH family members, found predominantly in the valve endothelial cells (Timmerman et al., 2004; MacGrogan et al., 2010) and members of the TGFB/BMP receptor families, found in the myocardium (Nakajima et al., 2000; Person et al., K005). Thus, is it unsurprising that many of the autosomal dominant gene variants, including *NOTCH1*, *JAG1*, *TGFB2*, *TGFBR1* and *SMAD3,* that are associated with congenital valve abnormalities have a role in these signaling pathways (Bravo-Jaimes and Prakash, 2020). While the role of WNT and VEGFA pathways to normal valve development has been known for some time, gene variants associated with these pathways are now being identified in polygenic valve diseases (Williams et al., 2019).

Both, NOTCH and TGFB/BMP signaling pathways regulate a number of transcription factors, such as GATA binding protein 4 (GATA4), snail family transcriptional repressor 1 (SNAI1), SRY-box transcription factor 9 (SOX9) and T-box transcription factor 20 (TBX20) which regulate the expression of genes required for the proliferation of newly transformed cells and remodeling of the matrix (MacGrogan et al., 2014; Bravo-Jaimes and Prakash, 2020). NOTCH1 and its downstream mediators have also been found to be expressed in an adult aortic valve and its loss has been associated with areas of calcification in human aortic valves (Mohamed et al., 2006). Whereas, expression of WNT5a and WNT11 is associated with BAV and calcification, and inhibition of these signaling pathways reduced mineralization in an *ex-vivo* model (Albanese et al., 2017). Further comprehensive analysis of these signaling pathways may help in identification of common signaling modules and provide a greater insight into the genetic basis of valve development.

Cardiac valves stratify into highly organized collagen, elastin and proteoglycan extracellular matrix (Rabkin-Aikawa et al., 2004). The development of a normal cardiac valve also requires specific structural proteins. Consequently, variants in the genes encoding structural proteins elastin (ELN), fibrillin 1 (FBN1) and collagen type III alpha 1 chain (COL3A1) have been associated with congenital valve defects. In addition, gene variants encoding proteins required to ensure specific localization of the cells through cell-cell and cell-matrix interactions, such as elastin microfibril interfacer 1 (EMILIN1), have been detected in patients with valve defects (Wu et al., 2017).

**FIGURE 1:**
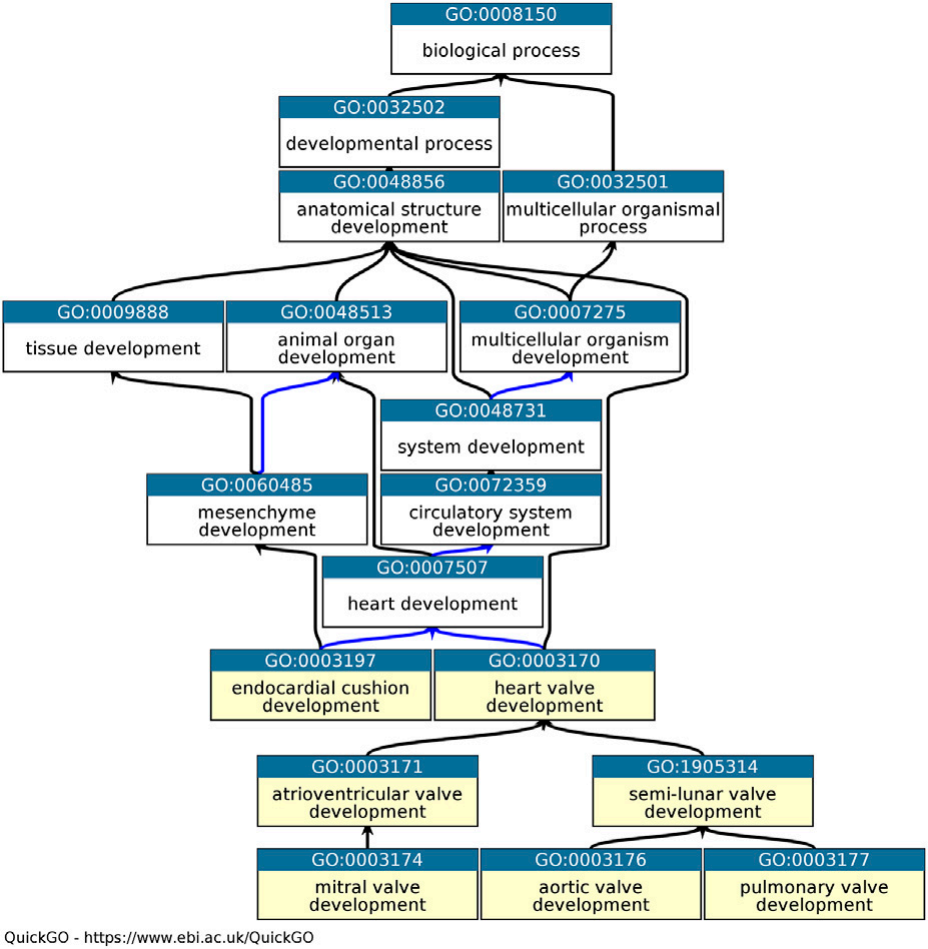
Ontology relevant to heart valve development. Graph of the part of the heart development ontology, available in QuickGO (Binns et al., 2009). Seven descriptive child terms of ‘*heart development*’ are highlighted in yellow. The ‘is_a’ relations between GO terms are indicated by black arrows, the ‘part_of’ relations as blue arrows (Ashburner et al., 2000; Gene Ontology Consortium, 2021).

In order to advance treatment of aortic valve disease it is necessary to study aortic valve development at both morphological and genetic levels to obtain a multifaceted understanding. The Gene Ontology (GO) is one of several bioinformatic resources which can contribute to the understanding of the genetic basis of aortic valve development. The GO Consortium provides a freely available resource with the goal of unifying the representation of genes and gene product attributes from the biological and biomedical literature (Ashburner et al., 2000; Gene Ontology Consortium, 2021). Three GO terms ‘*biological process*’, ‘*cellular component*’, and ‘*molecular function*’ form the ‘roots’ of the ontological tree. Although, the GO Consortium captures the normal roles of genes and their products in any organism, through structured and precise vocabulary, phenotype data can be used to support GO annotations. The tiered organization of GO terms within the ontology provides specific relations between descriptive child terms and the less descriptive parent terms (Figure 1). This ontology structure ensures that there is no loss of information when applying the specific GO terms. For example, ‘*aortic valve morphogenesis*’ (GO:0003180) is a child term of ‘*aortic valve development*’ (GO:0003176) which in turn is a child term of ‘*heart valve development*’ (GO:0003170). Consequently, a search for the term ‘*heart valve development*’ will retrieve these annotations, along with more specific GO term annotations. A basic GO annotation associates a GO term with a specific gene product. However, the annotation captured can be improved with additional contextual information included in the ‘annotation extension’ field (Huntley et al., 2014). The ‘annotation extension’ field allows the curator to add additional information such as the cell or anatomical location, by using Cell Ontology and UBERON terms (Bard et al., 2005; Mungall et al., 2012) or a regulated target, such as a gene whose expression is regulated by the annotated transcription factor (Huntley et al., 2014). For example, the mouse protein Rbpj is associated with the GO term ‘*positive regulation of transcription by RNA polymerase II*’ (GO:0045944) and the annotation extension ‘occurs_in’ ‘*aortic valve*’ (UBERON: 0002137), ‘has_input’ Tnf (Tumor necrosis factor, UniProt ID: P06804).

The British Heart Foundation (BHF) supported biocurators at University College London (UCL) previously organized the expansion of the heart development and cardiac physiology ontologies (Khodiyar et al., 2011; Lovering et al., 2018). Using these ontologies we have created over 2,000 GO annotations that describe heart development and cardiac physiology (Bard, 2005; Mungall et al., 2012; Lovering et al., 2018; Khodiyar and Lovering, 2013; Li et al., 2022; https://tinyurl.com/QuickGOheartBHF), of which around a fifth are relevant to normal heart valve development (https://tinyurl.com/QuickGOvalveBHF). These valve development annotations were created as part of a heart valve development annotation project focused on identifying the most specific and appropriate GO terms to associate with each protein record. For example, some of these annotations describe the role of proteins in early embryonic processes such as ‘*embryonic heart tube left/right pattern formation*’, ‘*outflow tract morphogenesis*’ and ‘*cardiac jelly development*’. The requirement of other proteins in later stages in heart valve development has also been captured by applying terms such as ‘aortic valve development’, ‘*pulmonary valve development*’ and ‘*mitral valve morphogenesis*’.

Prior to this work, the UCL team had focused on annotations relevant to developmental and pathological cardiovascular biology and, consequently, had identified a lack of GO annotation describing heart valve development (Khodiyar et al., 2011; Khodiyar and Lovering, 2013; Lovering et al., 2018; Li et al., 2022). Consequently, this heart valve development manual annotation project has exploited the GO to describing over 160 proteins associated with heart valve development. Our curation of published experimental evidence has led to the creation of 400 descriptive GO annotations describing the molecular contribution to heart valve development. However, around 600 of the 1,000 annotations created during this heart valve focused project captured the cellular role of these proteins, in other processes. Some of the applied terms captured processes that are required for normal heart valve, such as BMP signaling and endocardial cushion development, but some annotations were less relevant, such as aortic valve morphogenesis. In addition, we demonstrate how our contribution to the GO resource has improved interpretation of protein interaction networks. Furthermore, we discuss how this data can improve the analysis of GWAS, NGS and transcriptomic datasets aimed at investigating some of the most common congenital cardiac diseases.

## 2 Materials and methods

### 2.1 Selection of articles and curation

The priority list of proteins for annotation included 99 candidate BAV associated proteins, that had been generated by Bonachea et al. (2014), to use in a targeted capture strategy to identify BAV associated genes by NGS (Bonachea et al., 2014). This list was expanded to 138 valve development-associated proteins following a review of the literature, with a focus on NOTCH, TGFB and BMP signaling pathways (Supplementary Table S1). A literature search identified experimental articles that described the role of these priority proteins in heart valve development. Articles were prioritized for curation according to the following criteria: species (articles only curated if the species of the protein examined was identifiable), description of aortic valve development.

**FIGURE 2:**
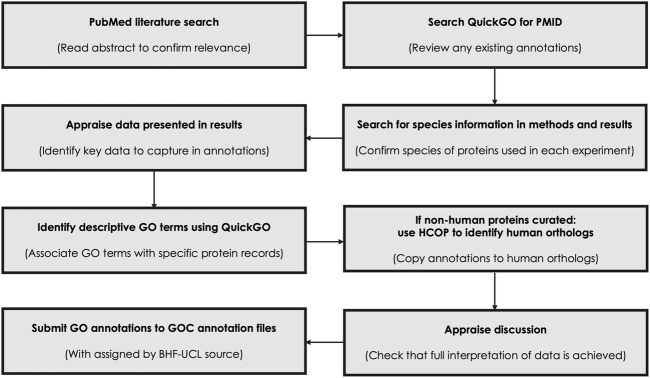
Flowchart highlighting the steps taken to manually curate each article. Manual GO curation of published articles applied GO Consortium guidelines and use the GO browser, QuickGO, to identify any previous annotations associated with each article as well as to identify appropriate GO terms to associate with the described proteins. The HGNC orthology prediction tool HCOP (Yates et al., 2021), was used to confirm orthology between non-human proteins and human proteins.

High quality manual GO annotations were created following the existing GO Consortium guidelines (Figure 2; Balakrishnan et al., 2013), these annotations were submitted to the GO annotation resource and made public with the assigned by source BHF-UCL (British Heart Foundation-University College London, Lovering et al., 2018). Annotation Extensions were associated, when possible, to provide more detailed information (Huntley et al., 2014). The following evidence codes were used: IDA—Inferred from Direct Assay; IMP—Inferred from Mutant Phenotype; IGI—Inferred from Genetic Interaction, with the interacting protein recorded in the ‘With’ field; IC—Inferred by Curator, only used where an annotation was not supported by any direct experimental evidence; ISS—Inferred from sequence or structural similarity, used to copy annotations associated with non-human species (mouse, rat, pig, zebrafish, chicken) to the human ortholog once 1-to-1 orthology between the species had been verified using the HGNC (HUGO Gene Nomenclature Committee) Comparison of Orthology Predictions (HCOP) tool (Yates et al., 2021).

### 2.2 Functional enrichment analysis

The Database for Annotation, Visualization and Integrated Discovery (Sherman et al., 2022) functional enrichment analysis tool was used to investigate the frequency of association of biological process GO terms with a list of 28 genes with exonic variants associated with BAV (Bonachea et al., 2014; Supplementary Table S1). HGNC approved gene symbols were used in the analysis, which was conducted on 15 April 2023. The default background set was used which included 19,357 human genes that were associated with at least 1 biological process GO term. Note that this analysis creates GO term groups which includes ‘regulation’ child term annotations, in addition to the ‘part_of’ and ‘is_a’ child term annotations.

## 3 Results

### 3.1 Identification of aortic valve development articles to annotate

The priority list of 138 valve development-associated proteins was generated using a previous list of 99 candidate BAV associated proteins (Bonachea et al., 2014) and a review of the literature, with a focus on NOTCH, TGFB and BMP signaling pathways (Supplementary Table S1). An extensive literature review led to the manual curation of 100 heart valve development-relevant articles (https://tinyurl.com/QuickGOBHFheartvalve, Supplementary Table S4, S5). During this review, articles that did not present experimental data or provided no new annotations were excluded. Occasionally, an article was curated that had previously been curated, however, this was only undertaken if key information describing valve development had not been captured. Time constraints prevented the curation of the role of all priority proteins. Full annotation of the selected articles led to the annotation of 211 proteins and the creation of 1,026 annotations. Although the project was focused on heart valve development, only 208 annotations applied GO terms specifically describing this process to 99 proteins. However, comprehensive annotation of each article, resulted in 329 annotations to earlier heart development terms, such as ‘*embryonic heart tube left/right pattern formation*’, ‘*endocardial cushion morphogenesis*’ and ‘*outflow tract morphogenesis*’, providing information for a further 71 proteins. It is important to capture these early developmental processes because when these do not proceed normally there can be abnormalities in valve development. The full annotation of each article added another 489 annotations, even though these additional annotations were not directly related to heart valve development.

The published experimental literature available on the subject of genetic pathways involved in aortic valve disease is mostly based on mouse models, and therefore, the majority of the annotations with experimental evidence codes were associated with mouse proteins rather than human proteins. In addition, articles describing the genes involved in heart valve development in rat, zebrafish and pig were also curated. These non-human annotations were then used to infer functionality of human proteins and were copied to the orthologous human protein records using the ISS-curator evidence code (Inferred by Sequence Similarity, Balakrishnan et al., 2013).

In order to measure the degree of annotation enrichment we calculated the number of human proteins associated with three key GO terms before the start (i.e., January 2012) and after completion (April 2023) of this project. The three terms selected were ‘*aortic valve development*’ (GO:0003176); its parent term, ‘*heart valve development*’ (GO:0003170); and, the term ‘*endocardial cushion development*’ (GO:0003197) which refers to the development of the key primitive structure that gives rise to heart valves during embryological life. In January 2012, there were only 16 human proteins associated with one of these GO terms, through manual annotation. By the end of this project, however, there were 74 human proteins associated with the term ‘*heart valve development*’, 53 associated with ‘*endocardial cushion development*’, and 43 associated with ‘*aortic valve development*’ (see Table 1). With over 90% of the manual annotations generated by this heart valve focused project.

**TABLE 1:** Summary of human heart valve annotation progress.

GO term ID		GO:0003176	GO:0003197	GO:0003170	
GO term name		Aortic valve development	Endocardial cushion development	Heart valve development	Total for 3 terms
pre-2012	Number of proteins manually curated	5	7	11	16
	Number of manual annotations	6	7	20	27
2023	Number of proteins manually curated	43	42	64	77
	Number of proteins manually curated by BHF-UCL	42	39	58	70
	Total number of proteins associated with term (including IEA)	43	53	74	94
	Number of manual annotations	54	76	135	211
	Number of manual annotations created by BHF-UCL	53	71	125	196
	Total number of annotations applying term (including IEA)	81	155	232	396

Number of GO annotations and human proteins associated with each term (or their child terms) before January 2012 and in April 2023, the totals at the bottom of the table include IEA (inferred by electronic annotation) annotations.^17,18^ Annotations to ‘*aortic valve development*’ are included in the ‘*heart valve development*’ total, due to the parent:child relation between these terms. Data downloaded from QuickGO,^22^ 13 April 2023 (https://tinyurl.com/QuickGOhumanHeartValve; Supplementary Table S2).

The 70 heart valve proteins curated by this project are involved in various early heart development signaling pathways, including BMP, NOTCH, WNT, SLIT/ROBO, Smoothened, TGFB and WNT. In addition, some of these curated proteins are structural constituents, such as cadherin 11 (CDH11) and elastin (ELN, Supplementary Table S2). A selection of GO annotations describing the role of various proteins are described below.

### 3.2 Proteins involved in the NOTCH signaling pathway

Due to the widespread role of the NOTCH1 signaling pathway and its role in the cardiovascular system, *NOTCH1* gene is one of the most widely studied genes in relation to valve development. In humans, *NOTCH1* variants are associated with BAV (Garg et al., 2005). Furthermore, mutations in other NOTCH signaling pathway proteins have been shown to cause aortic valve abnormalities (Williams et al., 2019). This project associated eleven NOTCH signaling pathway proteins, or proteins that regulate this pathway, with the GO terms, ‘*aortic valve development*’, ‘*heart valve development*’ and ‘*endocardial cushion development*’ as well as other GO terms (https://tinyurl.com/QuickGOhumanHeartValve, Supplementary Table S2).

A typical experiment that was curated during this project is provided by Wang et al., who demonstrated altered Notch signaling in the endothelial and interstitial cells of developing valves through use of mouse conditional models of *Notch1* mutant phenotypes (Wang et al., 2017). The article demonstrated that inactivation of *Notch1* in valvar endothelial cells resulted in a wide range of valvar and myocardial defects including enlarged semilunar valves, fibrotic valves, ventricular septal defects and hypertrophic left ventricle, providing experimental evidence that *Notch1* was involved in, ‘*aortic valve morphogenesis’* and ‘*pulmonary valve morphogenesis*’, amongst others; these processes were hence associated with the mouse Notch1 protein record and then copied, using the ISS evidence code to the human NOTCH1 protein record. Another example of data captured during this project is provided by, (Koenig et al., 2016; Koenig et al., 2016) Using *Notch1* +/−, *Nos3* −/− and *Notch1* +/−; *Nos3* −/− compound mutant mice, Koenig et al., 2016, studied the role of endothelial Notch1 and Nos3 in the development of semilunar valves and cardiac outflow tract using mice embryos (Koenig et al., 2016). Histological analysis and immunofluorescence of hearts obtained from these mice embryos exhibited thickened malformed semilunar (aortic and pulmonary) valves, defective endocardial cushion formation and ventricular septal defects, demonstrating the role that Notch1 with Nos3 had in normal aortic valve morphogenesis, pulmonary valve morphogenesis, endocardial cushion morphogenesis and ventricular septum morphogenesis (Koenig et al., 2016). This information was captured by associating the term ‘*endocardial cushion development*’ with both mouse Notch1 and Nos3 using the IGI evidence code due to their linked activities with one another. Again, these mouse protein annotations were copied to the human orthologous protein records.

### 3.3 Proteins involved in the BMP and TGFB signaling pathways

The BMP and TGFB family signaling pathways play a critical role in the regulation of cell growth, differentiation, and development in a wide range of biological systems (Miyazono, 2000). These pathways includes the BMP and TGFB family ligands, their receptors, the SMADs, which are responsible for intracellular signaling, and the transcription factors which regulate the expression of the signaling pathway’s target genes (Miyazono, 2000). The role of 28 proteins involved in BMP and TGFB signaling pathways were curated during this project. (https://tinyurl.com/QuickGOhumanHeartValve, Supplementary Table S2).

GATA binding protein 4 (GATA4) is zinc-finger transcription factor that acts downstream of many TGFB signaling pathways. The important role of GATA4 in aortic valve development was confirmed by Li et al., 2018, following direct sequencing of GATA4 in 150 patients with congenital BAV (Li et al., 2018). This evidence supported the association the human GATA4 protein record with the term ‘*aortic valve morphogenesis*’. Similar to GATA4, the importance of GATA5 in normal aortic valve development has also been demonstrated by the association of *GATA5* variants with BAV (Shi et al., 2014). Shi et al. genotyped *GATA5* in patients with BAV*,* and using a luciferase reporter assay system, characterized the functional effect of the mutations (Shi et al., 2014). This provided evidence that variations in *GATA5* transcriptional activation domains may play a role in development of BAV in humans. Laforest et al., showed that murine Gata5 is required for normal endocardial cushion fusion (Laforest et al., 2011), this information was captured using the GO term ‘*endocardial cushion fusion*’. Additional contextual information was included in the GO annotations associated with Gata5 to capture its role, as a DNA binding transcription factor, positively regulating the expression of multiple genes, including *Bmp4, Cdh5* (cadherin 5), *Jag1* (jagged canonical Notch ligand 1), *Notch1*, and T-box 20 (*Tbx20*).

### 3.4 Functional enrichment analysis

After Bonachea et al. (2014) had identified 28 genes with variants associated with BAV they used the functional enrichment tool DAVID (Sherman et al., 2022) and identified that the WNT signaling pathway was associated with this gene list (Bonachea et al., 2014). In order to investigate whether the heart valve project annotations had changed the utility of GO, the same gene list was analysed again using DAVID. This analysis identified 879 enriched GO terms and revealed the relevance of several other signaling pathways with the development of BAV (Table 2; Supplementary Table S3). 26 of the 28 genes in this list are associated with the GO term ‘*signaling*’. In addition to enrichment of the GO term ‘*Wnt signaling pathway*’, this new analysis also identifies Notch, BMP, smoothened, vascular endothelial growth factor (VEGF) signaling pathways. The DAVID analysis also identified that there is experimental evidence to support the association of 11 of the 28 genes with the GO term ‘*heart valve development*’.

**TABLE 2:** Enrichment analysis of 28 genes with variants associated with BAV.

GO term name	Count	Fold enrichment	Pop hits	*p*-value	Genes
Enriched terms in the signaling domain					
signaling	26	2.66	6,757	5.64E-10	NOTCH2, SLC35B2, NOTCH3, FLT1, NOTCH1, GATA5, GATA4, GLI1, PPP3CA, SOX9, MSX1, NOS1, WNT4, JAG1, PTCH1, PTCH2, AXIN1, VEGFB, VEGFC, PAX6, NFATC1, AXIN2, TBX5, APC, SNAI1, MCTP2
Wnt signaling pathway	8	11.38	486	3.46E-06	NOTCH1, APC, AXIN1, NFATC1, SOX9, AXIN2, GLI1, WNT4
Notch signaling pathway	7	27.03	179	1.45E-07	NOTCH2, NOTCH3, NOTCH1, JAG1, SNAI1, GATA5, SOX9
BMP signaling pathway	4	15.54	178	1.90E-03	NOTCH2, NOTCH1, GATA4, MSX1
calcium-mediated signaling	4	12.86	215	3.25E-03	PPP3CA, NFATC1, NOS1, MCTP2
smoothened signaling pathway	4	20.48	135	8.58E-04	PTCH1, PTCH2, PAX6, GLI1
vascular endothelial growth factor signaling pathway	3	44.13	47	1.95E-03	FLT1, VEGFB, VEGFC
calcineurin-NFAT signaling cascade	2	32.15	43	5.83E-02	PPP3CA, NFATC1
inositol phosphate-mediated signaling	2	25.14	55	7.40E-02	PPP3CA, NFATC1
Enriched terms relevant to heart valve development					
heart valve development	11	104.17	73	2.44E-18	NOTCH2, NOTCH1, JAG1, APC, SNAI1, GATA5, GATA4, NFATC1, SOX9, AXIN2, TBX5
semi-lunar valve development	9	132.38	47	1.38E-15	NOTCH2, NOTCH1, JAG1, SNAI1, GATA5, GATA4, NFATC1, SOX9, AXIN2
endocardial cushion development	9	119.65	52	3.28E-15	NOTCH1, JAG1, APC, SNAI1, GATA5, GATA4, SOX9, MSX1, TBX5
aortic valve development	8	128.62	43	1.37E-13	NOTCH1, JAG1, SNAI1, GATA5, GATA4, NFATC1, SOX9, AXIN2
pulmonary valve development	4	120.23	23	4.21E-06	NOTCH2, NOTCH1, JAG1, NFATC1
atrioventricular valve development	4	98.76	28	7.75E-06	NOTCH1, GATA4, AXIN2, TBX5
mitral valve development	2	115.22	12	1.66E-02	NOTCH1, AXIN2

A functional enrichment of the 28 genes with exonic variants associated with BAV identified by Bonachea et al. (2014), was conducted using the DAVID enrichment tool (Sherman et al., 2022) on 15 April 2023 (Supplementary Table S3). The Count column indicates the number of genes associated with the GO term that are also in the analysed list. Pop Hits list the number of background genes associated with the GO term. The Fold Enrichment and *p-*value were provided by DAVID. The Genes column provides the names of the genes analysed associated with the enriched GO term.

## 4 Discussion

Aortic valve disease is one of the most common forms of congenital heart disease (CHD) in the world (Bonachea et al., 2014) Understanding the developmental processes and genetic influences responsible for this disease is crucial in order to improve treatments and increase longevity of these patients (Batsis and Lopez-Jimenez, 2010). Congenital aortic valve disease is often indicative of underlying genetic factors that are responsible for altering normal aortic valve development, including over-expression and under-expression of specific genes (Wirrig and Yutzey, 2014; Wu et al., 2017; Williams et al., 2019).

We have identified and developed a previously under-annotated area of the GO concerning the proteins involved in aortic valve development and, by extension, heart valves in general. Our annotations were predominantly assigned to mouse proteins with direct experimental evidence and then copied to the human orthologs. The close evolutionary conservation of mammalian heart development allows for the identification of proteins that are likely to have shared roles between species.

The value of mouse model data is demonstrated by our DAVID analysis which identified that 11 of the 28 genes with exonic variants in BAV patients (Bonachea et al., 2014) were associated with the GO term ‘*heart valve development*’. The majority of these annotations will have been created using mouse models. The association of the human gene orthologs with congenital BAV risk demonstrates the value of mouse model data in human disease models. This also confirms that the data within the GO resource can be used for clinical research, for example, the extraction of candidate risk factors for human congenital valvar disease.

Following the curation of 100 heart valve development articles, 174 proteins across 9 species, including human, mouse, zebrafish, pig, chicken, and zebrafish, were annotated, creating over 1000 GO annotations (Supplementary Table S5). The majority of the annotated proteins, were mouse (88 proteins) or human (65 proteins). Many of these proteins have a role in NOTCH, BMP, TGFB or WNT signaling pathways, and over half of the annotations described either heart development or a blood vessel development term (436 and 89 annotations respectively), with 71 describing heart valve development and 75 describing endocardial cushion development. Despite the focus of this project on heart valve development, 144 of this project’s annotations, applied GO terms describing other developmental processes. This was due to the versatile and widely-observed roles of the curated proteins in the development of other tissues. Whilst full article curation increased the time taken to annotate heart valve development, annotation of all available evidence in the selected literature assists in describing the mutual pathways within various developmental processes. Thus, full article curation increases the utility of the GO as an in-depth and descriptive resource with a breadth of coverage at both gene and knowledge levels. This not only feeds into the principal goal of the GO Consortium as a universal repository for functional biological knowledge, but is also of benefit to researchers using GO (Lovering et al., 2018).

Prior to this project, there were only 6, 20 and 7 human proteins manually associated with the GO terms ‘*aortic valve development*’, ‘*heart valve development*’ and ‘*endocardial cushion development*’, respectively. The number of proteins currently associated with these terms (43, 74 and 76, Table 1) confirms the impact of this project on populating the GO resource with annotations. The GO terms associated with these proteins now provides a more accurate reflection of the current state of understanding about the roles of various signaling pathways and proteins involved in the development of the human heart valves.

The original functional enrichment analysis of the 28 BAV-associated genes identified only the WNT signaling pathway contributing to this disease. Although this article was published in 2014, the functional analysis tool DAVID was using the GO data released in 2009. Our analysis, using the GO data released in 2023, identified 8 specific signaling pathways, including WNT signaling, but also adding NOTCH, BMP, calcium-mediated, smoothened and VEGF. Multiple, cross-regulating, signaling pathways are known to be required for normal heart valve development. Thus, our full article approach to annotation of heart valve developmental processes, along with the annotations submitted by other members of the GO consortium, has enabled a more appropriate interpretation of Bonachea et al.’s data (Bonachea et al., 2014).

The association of the GO term ‘*heart valve development*’ with over 70 proteins can now be used as a resource for next-generation sequencing (NGS) analysis of patients with AVD (or other valve diseases). By targeted sequencing of these genes, or by using these annotations to filter the identified variants in a patient, it may be possible to identify more likely candidate genes contributing to disease. The majority of NGS analysis tools currently use mouse phenotype (MP) data, or the Genomics England PanelApp data from the 100,000-genome project (Martin et al., 2019). However, only three of the 42 proteins associated with the GO term ‘*aortic valve development*’, ELN, NOTCH1, and SMAD6, have been identified by the Genomics England PanelApp to be involved in aortic valve disease (AVD). Thus, the annotations created by this project can be used by other biocurators to ensure the incusion of many more heart valve disease associated proteins in genomic databases, such as the Genomics England PanelApp.

In addition, as the majority of articles studied in this project were those describing mouse models it might be expected that MP annotations which are based on mouse mutant studies (Eppig, 2017) will be a better source of data than GO. A search for the mouse genes associated with the MP term ‘*abnormal heart valve morphology*’ (MP:0000285), identified 262 genes which have the potential to disrupt normal heart valve development. 51 of the 64 human genes manually associated with the GO ‘*heart valve development*’ (or child) term are also associated to this ‘equivalent’ MP term, leaving 13 of the GO annotated genes not associated with the MP ‘*abnormal heart valve morphology*’ term. This suggests that the GO needs to associate at least 211 additional genes to the ‘*heart valve development*’ (or child) term and that MP might be able to associate a further 13 genes to the ‘*abnormal heart valve morphology*’ term.

A further interesting field for annotation would be the role of the various signaling pathways in adult heart valve disease. So far, our understanding of valve disease presented in adults is limited based on the etiology; congenital or acquired. The genetic basis of adult valve disease still remains unchartered territory with immunogenicity being blamed as the main culprit for calcification. However, as NOTCH1, RB1 and TGFB1 not only play a role in the development of aortic valve, but also appear to prevent valve calcification, it seems likely that other processes may underlie this condition. An understanding of the role of signaling pathways involved in adult valve disease could be achieved by comparing the transcriptome or proteome of explanted calcified valves from adult patients undergoing aortic valve replacement surgery, with healthy aortic valve transcriptome or proteome data. This may lead to the identification of additional genes involved in adult valve calcification and consequently support the development of targeted therapies to combat this disease.

### 4.1 Conclusion

We have captured the role of BMP, NOTCH, TGFB and other signaling pathways in heart valve development using GO annotations. These annotations can be included in statistical analysis of heart disease associated datasets, using the freely available GO resource. Due to time constraints, we were unable to curate every protein that contributes to heart valve development and the associated signalling pathways. Instead, through this project, we have created a list of proteins across various signalling pathways that can be used as the primary resource for researchers investigating proteins with a role in heart valve development. Completing the annotation of all gene products, proteins and non-coding RNAs, that are required to ensure normal heart valve development remains an outstanding aim. Future GO curation projects could use our list of 138 priority proteins, in addition to the list of proteins associated with the MP term ‘*abnormal heart valve morphology*’ to complete curation of heart valve development. This would provide researchers and clinicians an expanded substrate for heart valve disease screening and directing further research towards gene targeted therapies for treatment of these diseases.

The future utility of the GO resource depends on updates and refinements such as those carried out in this project to maintain a current and broad-scoped resource. With continued collaboration between bioinformaticians, basic scientists, and clinicians, these incremental improvements in understanding may eventually help develop a better understanding of valve diseases and improve the therapeutic outcomes for the affected patients.

## Data Availability

The datasets presented in this study can be found in online repositories. The names of the repositories can be found in the article and Supplementary Material. The annotations created by this project are freely available to download or browse using the GO browsers AmiGO2 (Gene Ontology Consortium 2021, http://amigo.geneontology.org/amigo/) filtering on Contributor ‘BHF-UCL’ or QuickGO (Binns et al., 2009, https://www.ebi.ac.uk/QuickGO/) filtering on gene products ‘UniProtKB, Reviewed (Swiss-Prot)’ and More > Assigned by ‘BHF-UCL Cardiovascular Gene Ontology Annotation Initiative’, or to download by ftp from ftp://ftp.ebi.ac.uk/pub/databases/GO/goa/.
